# A Longitudinal Pilot Study on Cognition and Cerebral Hemodynamics in a Mouse Model of Preeclampsia Superimposed on Hypertension: Looking at Mothers and Their Offspring

**DOI:** 10.3389/fphys.2021.611984

**Published:** 2021-02-01

**Authors:** Lianne J. Trigiani, Clotilde Lecrux, Jessika Royea, Julie L. Lavoie, Frédéric Lesage, Louise Pilote, Edith Hamel

**Affiliations:** ^1^Laboratory of Cerebrovascular Research, Montreal Neurological Institute, McGill University, Montréal, QC, Canada; ^2^Centre de Recherche du Centre Hospitalier de l’Universite de Montreal and School of Kinesiology and physical activity sciences, Université de Montréal, Montréal, QC, Canada; ^3^Biomedical Engineering Institute, École Polytechnique de Montréal, Montréal, QC, Canada; ^4^Department of Medicine, Centre for Outcomes Research and Evaluation, McGill University Health Centre, Montréal, QC, Canada

**Keywords:** preeclampsia, cerebrovascular function, cognition, renin—angiotensin—aldosterone system, cerebral hemodynamics

## Abstract

Preeclampsia is a common hypertensive disorder in pregnant women and whose causes and consequences have focused primarily on cardiovascular outcomes on the mother and offspring, often without taking into consideration the possible effects on the brain. One possible cause of preeclampsia has been attributed to alterations in the renin-angiotensin system, which has also been linked to cognitive decline. In this pilot study, we use a transgenic mouse model that chronically overexpresses human angiotensinogen and renin (R^+^A^+^ mice) that displayed characteristics of preeclampsia such as proteinuria during gestation. Offspring of these mothers as well as from control mothers were also examined. We were primarily interested in detecting whether cognitive deficits were present in the mothers and offspring in the long term and used a spatial learning and memory task as well as an object recognition task at three timepoints: 3, 8, and 12 months post-partum or post-natal, while measuring blood pressure and performing urine analysis after each timepoint. While we did not find significant deficits in preeclamptic mothers at the later timepoints, we did observe negative consequences in the pups of R^+^A^+^ mice that coincided with hemodynamic alterations whereby pups had higher whisker-evoked oxygenated hemoglobin levels and increased cerebral blood flow responses compared to control pups. Our study provides validation of this preeclampsia mouse model for future studies to decipher the underlying mechanisms of long-term cognitive deficits found in offspring.

## Introduction

Preeclampsia is a common hypertensive disorder in pregnant women, with a worldwide incidence rate of just under 3% but varying between 2.7 and 8.2% depending on the region studied (Kongwattanakul et al., 2018) and this incidence rate continues to grow (Fox et al., 2019). Considering these statistics, and despite the connection between hypertension and cognitive impairment and dementia (Obisesan, 2009; Walker et al., 2017; Wright and Harding, 2019), little is known about the long-term neurological consequences of preeclampsia on both mothers and even less so in children. What is well known in the preeclampsia field is the increased risk of cardiovascular incidents in mothers, including increased risk of long-term hypertension and stroke, and increased mortality due to major cardiovascular events, as well as early onset hypertension, increased risk of ischemic heart disease and strokes in children (Bokslag et al., 2016; Goffin et al., 2018). The focus of studies evaluating the risks associated with preeclampsia has thus been on cardiovascular health, often disregarding the relationship between cardiovascular disease and late-life dementia (Justin et al., 2013). Cognitive effects of preeclampsia on mothers revealed only subjective memory complaints in the short term with no significant long-term consequences after adjusting for other relevant risk actors, while developmental and behavioral consequences in offspring are evident, but not often studied beyond the age of 12 years old, again focusing mostly on short-term effects (Figueiro-Filho et al., 2017; Dayan et al., 2018; Elharram et al., 2018).

In part due to a lack of understanding underlying the etiology of preeclampsia and its multifactorial nature, different animal models have been used to mimic different aspects of this condition. These models recapitulate alterations in angiogenesis, immune responses, disrupted oxygen metabolism, and abnormal trophoblast invasion seen in humans (Pennington et al., 2012; Marshall et al., 2018). Of interest, the renin-angiotensin system (RAS) has been implicated in the development of preeclampsia (Shah, 2005) and alterations in the RAS have been linked to cognitive decline and dementia (Wright and Harding, 2019). Here, we used transgenic female mice overexpressing human angiotensinogen and renin (R^+^A^+^ females) that are chronically hypertensive. This is a realistic disease model of preeclampsia superimposed on chronic hypertension, not only because chronic hypertension represents a major risk factor for developing preeclampsia during pregnancy, but because these mice mimic several aspects of preeclampsia, including aggravated hypertension during pregnancy, glomerular endotheliosis, proteinuria, placental necrosis, lower pup weights, impaired angiogenesis of uteroplacental vessels, and increased circulating anti-angiogenic factors (Falcao et al., 2009; Marshall et al., 2018). This pilot study aimed to assess whether preeclampsia incurs any behavioral consequences in the long term in R^+^A^+^ mothers, and whether this is also imparted on their offspring. Our study corroborates findings from human studies and reveals the presence of cognitive impairment later in life in offspring born from preeclamptic mothers, thus providing further validation of this mouse model for future studies to decipher the underlying mechanisms of these cognitive deficits in mothers and offspring.

## Materials and Methods

### Study Design

Mice were produced by breeding transgenic male mice heterozygous for human renin (Ren9 line) with female mice heterozygous for overexpressing human angiotensinogen (204/1 line) (Falcao et al., 2009). An equal number (*n* = 15/group, 4 months of age) of control females (a mixture of R^–^A^–^, R^–^ A^+^, and R^+^A^–^ genotypes) and transgenic females with the R^+^A^+^ genotype, predisposed to become preeclamptic during pregnancy, entered the study. Baseline blood pressure (BP) was measured, and urine samples collected to assess proteinuria prior to mating. All females were then paired with a wild type (C57BL/6 background) male for breeding, and a female was deemed pregnant by the presence of a vaginal plug and later confirmed by weight gain. After 2 months of mating 13/15 control females became pregnant (average litter size of 5.69 ± 0.47), while only 8/15 R^+^A^+^ were pregnant (average litter size of 6.38 ± 0.65). Fifteen and eighteen days following the initial plug, a urine sample was collected, and BP was measured; this was also performed 5 days after giving birth, at which point pups were weighed. Urine samples and BP were taken in mothers 3, 6, and 12 months postpartum (PP). Cognitive testing on mothers was performed at 9, 14, and 18 months of age (or 3, 8, and 12 months PP) alongside a subset of their offspring (3, 8, and 12 months of age, equally distributed males and females). Likely due to R^+^A^+^ females being chronically hypertensive, we found a higher death rate over time in this group, leaving us with *n* = 5 at study endpoint (12 months PP). Due to differences in cognitive function in offspring at 12 months of age and sufficient group sizes, we used optical imaging of intrinsic signals (*n* = 6/group) to asses cerebral hemodynamic function. Experiments were approved by the Animal Ethics Committee of the Montreal Neurological Institute (McGill University, Montréal, Québec, Canada) and complied with the Canadian Council on Animal Care.

### Assessing Preeclampsia Traits in Mouse Model

#### Proteinuria

Urine samples (∼100 μL) were collected in 1.5 mL centrifuge tubes from mothers prior to being impregnated (baseline), and then after 15 days of gestation (day 1 defined by the presence of a plug), 18 days of gestation, 5 days PP and before the first behavioral test, 3 and 8 months PP. Samples were kept at −80°C, and then thawed and diluted 1:10 before measurements were made in duplicate. Proteinuria was determined as the ratio of albumin:creatinine using Albuwell and Creatinine companion mouse ELISA kits (Exocell, Philadelphia, PA) according to manufacturer’s protocol.

#### Blood Pressure (BP)

Blood pressure was measured (*n* = 10 mice/group) after each behavioral timepoint in mothers, and at 6 months of age in a subset of offspring using non-invasive tail-cuff plethysmography (Kent Scientific Company). Mice were habituated to the restraining device and tail cuffs for 10 min/d for 3 days before measurements were recorded. Ten additional acclimation cycles were performed before acquiring five measurements of systolic, diastolic, and mean blood pressure. This was performed 1 week prior to each behavioral testing session.

#### Pup Weight

Five days after birth, pups were individually weighed on a scale tarred with a small piece of nesting material and immediately returned to their mother’s nest. The average weight of the pups from individual mothers was calculated, and then compared based on mother’s genotype.

### Morris Water Maze (MWM)

Spatial cognitive function was evaluated in the MWM (Deipolyi et al., 2008) at 2 time points in mothers and offspring. A pool (1.4 m diameter) was filled with water (18 ± 1°C) made opaque with inert white Tempera paint. Three initial visible platform training days were performed: On each day, the mice were given three trials of 60 s separated by approximately 45 min to find the platform (15 cm diameter). Before the 5-days learning phase of the task began, the visual cues in the room were changed, as was the location of the platform, which was completely submerged (1 cm below the surface of the water). Mice were given three trials of 90 s to find the hidden platform. On the final day (day 9 for first timepoint), a probe trial was conducted to assess spatial memory. The platform was removed from the pool and the mice were given 60 s of free swim in which the time they spent swimming and the distance they swam in the quadrant that contained the platform was recorded as well as the number of times they would have landed on the platform if it were present. All data was recorded using the 2020 Plus tracking system and Water 2020 software (Ganz FC62D video camera; HVS Image, Buckingham, United Kingdom). When the MWM was conducted at the second time point (8 months postpartum or 8 months of age for the pups), the only differences in the test were that the mice were only provided with one training day to locate the platform before it was hidden (the probe trial being performed on day 7), and the locations of the spatial cues and platform were changed. Due to the sample size of R^+^A^+^ mothers decreasing to *n* = 5 at 12 months PP, along with no cognitive deficits observed at 8 months PP, no MWM was conducted at this last timepoint.

### Novel Object Recognition (NOR)

Following the MWM, mothers and offspring were habituated (10 min) to the NOR testing arena (45 cm wide × 45 cm long × 50 cm high). The following day, mice underwent both the familiarization and test phases. During familiarization, each mouse was given 5 min to explore 2 identical objects placed in opposite corners of the arena, equidistant from the center. Two hours after familiarization, one of the objects was replaced with a novel object and another 5 min exploration period was allotted. Both the location of the novel object and the objects were counterbalanced. Behavior was recorded using iSpy software and ODLog to record the amount of time spent exploring each object. An investigation ratio was calculated by dividing the time spent with the novel object by the total time investigating both objects during the test phase. This test was conducted at 3 separate time points using different pairs of objects at each time.

### Imaging of Optical Intrinsic Signals (OIS)

A subset of pups (*n* = 6/group, 12 months) were implanted with a cranial window made of a cover glass glued on a thinned bone preparation over the left barrel cortex and allowed 2 weeks to recover before imaging. Mice were anesthetized (ketamine 85 mg/kg/xylazine 3 mg/kg, i.m.) and fixed with ear bars on a physiological platform (Harvard Apparatus #75–1,500, Saint-Laurent, QC, Canada) with online recording of body temperature, heart rate and respiratory rate. The platform was then moved to the imaging system (OIS200 from LabeoTech, Montréal, QC, Canada); the mouse cranial window placed parallel to the camera. Whisker-evoked changes in total, oxy- and deoxyhemoglobin (HbT, HbO, and HbR, respectively) were measured with three light emitting diodes (LEDs, 525, 590, and 625 nm) placed 8 cm from the cranial window. Whisker-evoked changes in cerebral blood flow (CBF) were measured with laser speckle contrast imaging using a 780 nm laser diode. Images were acquired at a frame rate of 30 Hz in total (7.5 Hz for each wavelength), with a 14 ms exposure time. Each recording consisted of 7 blocks of right whisker stimulation (8 Hz, 10 s followed by a rest period of 40 s using a piezo actuator). We included a jitter of 3 s to avoid habituation to the stimulation timing for the mice. Data were lowpass filtered at 0.3 Hz to suppress high frequency noise (mouse respiration) and analyzed using MATLAB to obtain whisker-evoked changes in cerebral blood volume and CBF from baseline. Analysis was as described in Dubeau et al. (2011). Briefly, reflectance signals were converted to changes in absorption ΔA = log(R/R_0_), and a pseudo-inverse and the modified Beer–Lambert law were used to extract relative changes in HbR and HbO. CBF was computed by quantifying the spatial contrast during laser illumination, defined as the ratio of the standard deviation to the mean intensity in a given spatial area. For each animal, the spatial location of the response in the contralateral barrel cortex was first identified. A region of interest around the maximum response was manually delineated avoiding medium, large arteries, and veins, and the signal averaged over all pixels to recover an impulse response function for each component: HbO, HbR, and CBF.

### Statistical Analysis

All behavioral and imaging experiments were performed blind to the identity of the mice. Independent samples *t*-tests were conducted using GraphPad Prism 7, a *p* = 0.05 was considered statistically significant.

## Results

### Model Characterization of Mothers

**FIGURE 1:**
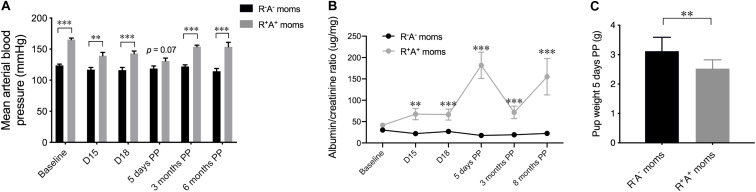
Preeclampsia model characteristics. **(A)** Mean arterial blood pressure shown at different timepoints for the mothers throughout the study beginning at baseline prior to mating, two times during pregnancy and three postpartum (PP) timepoints, revealing that this model is chronically hypertensive. **(B)** Urinalysis quantification over the same timepoints in mothers revealing an increased albumin:creatine ratio in R^+^A^+^ mothers during pregnancy that persisted up to 8 months PP. **(C)** The average pup weight 5 days PP from individual mothers was averaged and shown here indicating R^+^A^+^ birthed smaller pups than control mothers. *N* = 10–13 for control mothers and *n* = 5–8 for preeclamptic (R^+^A^+^) mothers, **p < 0.01 and ***p < 0.001.

Due to chronic overexpression of human angiotensinogen and renin, the mouse model used in this study of preeclampsia superimposed on chronic hypertension, is not only hypertensive during gestation, with a slight increase in BP from D15 to D18, but is chronically hypertensive as shown by elevated mean BP prior to pregnancy (baseline) and continued elevated BP levels when measured PP (Figure 1A). The majority of offspring (19/23) were negative for human renin and/or angiotensinogen, however 4 pups had the R^+^A^+^ genotype and were hypertensive when measured at 6 months of age (mean BP of 160.36 mmHg ± 3.2 compared to control pups 124.30 mmHg ± 3.49). These hypertensive pups were examined with caution following all measures, but they did not significantly differ from normotensive pups birthed by the same R^+^A^+^ mothers. A cardinal feature of preeclampsia is the presence of proteinuria during pregnancy, assessed here by the ratio of albumin to creatine (Figure 1B), which not only showed increases during gestation (D15 *p* < 0.01, and D18 *p* < 0.001) but also PP, spiking sharply at 5 days PP (*p* < 0.001) and remaining elevated at 3 months PP (*p* < 0.01), and even more so at 8 months PP (*p* < 0.001), prolonged or persistent proteinuria has been found as long as 2 years after delivery in preeclamptic women (Berks et al., 2009; Unverdi et al., 2013). Another indication of preeclampsia is a lower birth weight of the offspring, measured here 5 days PP, that revealed pups from preeclamptic mothers weighing significantly less (*p* < 0.01) than those from control mothers (Figure 1C).

### Behavioral Consequences on Mothers and Offspring

**FIGURE 2:**
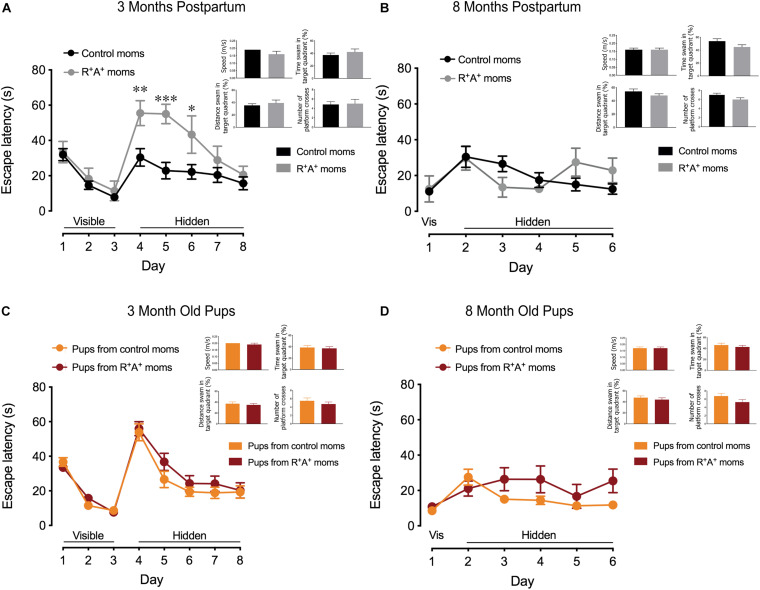
Spatial learning and memory testing in mothers and offspring. **(A)** Spatial learning portion of MWM in mothers 3 months PP showing deficits in preeclamptic mothers (R^+^A^+^, *n* = 7) early during the learning phase but without any spatial memory deficits measured during the probe trial compared to control mothers (*n* = 12). **(C)** Regardless of the genotype of their birth mother, 3-months old pups (*n* = 18–26/group) showed no differences in spatial learning nor memory. **(B)** At 8 months PP another MWM was conducted, revealing no spatial learning and memory deficits in the mothers nor in the pups **(D)**. Sample size at 8 months PP, control mothers *n* = 10, R^+^A^+^ mothers *n* = 6. Due to no observed sex differences in pups at 3 months of age, group sizes were reduced at random when tested at 8 months to *n* = 11/group. *p < 0.05, **p < 0.01, and ***p < 0.001.

**FIGURE 3:**
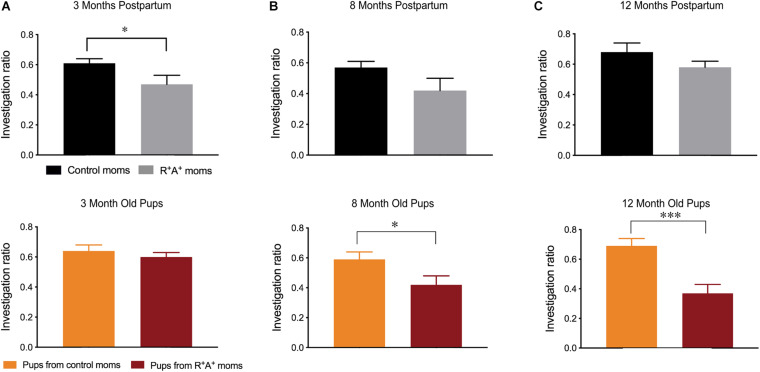
Novel object recognition memory testing in mothers and offspring. **(A)** NOR investigation ratios for mothers 3 months PP (R^+^A^+^ *n* = 7, controls *n* = 12) and pups at 3 months of age (*n* = 18–26/group) revealing a deficit in preeclamptic mothers. While no deficits were observed in mothers at 8 **(B)** nor 12 months PP **(C)**, pups born from preeclamptic mothers had impaired performance at these two age points **(B,C)**. Samples sizes at 8 and 12 months: control mothers *n* = 10, R^+^A^+^ mothers *n* = 5–6, pups *n* = 11/group. *p < 0.05 ***p < 0.001.

When tested on the MWM for spatial learning and memory, a primarily hippocampal-dependent task, preeclamptic mothers did not differ from control mothers on the visible platform training days (days 1–3) 3 months PP, indicating that they were capable of performing the task. However, when the platform was hidden, preeclamptic mothers took significantly more time to locate the platform on days 4–6 (Figure 2A) before learning the location to the same capacity as the control mothers on days 7 and 8, where there is no difference in escape latency. On day 9 when assessed for spatial memory, there were no differences between groups on any parameter measured (Figure 2A). Interestingly, the differences observed in spatial learning did not persist at 8 months PP in the mothers, and no difference in spatial memory was observed (Figure 2B). When comparing the pups of these mothers on the same MWM task at both 3 and 8 months of age, no differences were observed in spatial learning nor in spatial memory (Figures 2C,D). When testing for object recognition memory at 3 months PP in the dams and 3 months of age for the pups (Figure 3A), only preeclamptic mothers showed a deficit (*p* < 0.05). Despite a trend (*p* = 0.09) this deficit was no longer present at 8 months PP (Figure 3B) nor at 12 months PP in mothers (Figure 3C). However the pups from preeclamptic mothers presented a different tendency such that they began to display a significant deficit on this object memory task (investigation ratio of 0.42 ± 0.06 compared to 0.59 ± 0.05 in controls, *p* < 0.05) at 8 months, and not only did this deficit persist but also worsened when tested at 12 months of age (investigation ratio of 0.37 ± 0.06 compared to 0.69 ± 0.05 in controls, *p* < 0.001).

### Hemodynamic Alterations in Offspring

**FIGURE 4:**
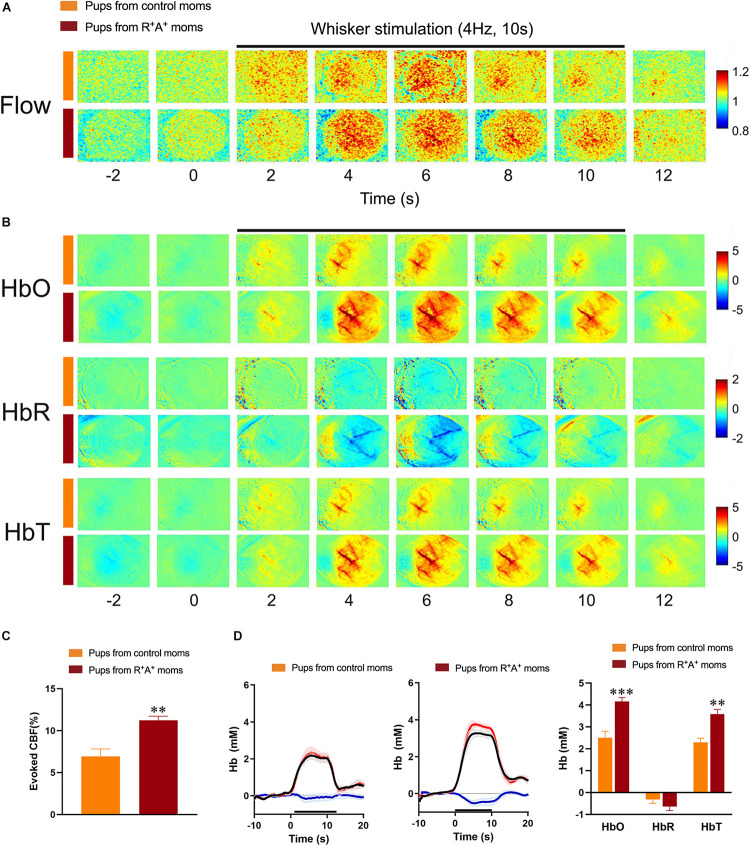
Evaluation of hemodynamic alterations in offspring at 12 months of age. **(A)** Functional laser speckle contrast imaging maps representative of cerebral blood flow (CBF) changes in the barrel cortex in response to whisker stimulation. **(B)** Functional imaging maps of oxygenated (HbO), deoxygenated (HbR) and total hemoglobin (HbT) levels from 4 s prior to stimulus to 2 s after a 10 s whisker stimulation (4 Hz) in offspring of preeclamptic (red bars) and control mothers (orange bars). **(C)** Quantification of laser Speckle contrast imaging showing an increased evoked CBF response in pups from preeclamptic mothers. **(D)** Average traces and quantification of peak concentrations (mM) of HbO (red line), HbR (blue line), and HbT (black line) from offspring revealing a significant increase in HbO and HbT levels in pups from preeclamptic mothers. *N* = 6/group **p < 0.01 and ***p < 0.001.

Due to the presence of a cognitive deficit in the pups at the 12-months endpoint, offspring were examined for possible hemodynamic alterations that could be contributing to worse cognitive performance. Surprisingly, we found there was a larger whisker-evoked CBF response in the contralateral barrel cortex (11.22% ± 0.49 increase from baseline) in pups from preeclamptic mothers compared to that elicited in the offspring of control mothers (6.93% ± 0.89, Figure 4A) and quantified in Figure 4C. In line with this finding, whisker stimulation elicited an enhanced response in total hemoglobin (HbT) during the stimulation period [*t*(10) = 4.51, *p* < 0.01] in the offspring of preeclamptic mothers, largely driven by an increase in oxygenated Hb (HbO) by nearly twofold: HbO during whisker-stimulation of pups from preeclamptic mothers being 4.16 mM ± 0.17, and 2.50 mM ± 0.30 from control pups *t*(10) = 4.22, *p* < 0.01. No differences in deoxygenated Hb (HbR) were observed between groups. These findings are consistent with whisker-evoked increases in CBF driving the surge in HbO, corresponding to fresh supply of arterial blood, with HbR, typically in veins, being washed out of the activated region, as illustrated by the decreased HbR (Figure 4B for raw images and Figure 4D for traces and quantification) (Ma et al., 2016).

## Discussion

In this pilot study, we investigated the long-term cognitive consequences in both preeclamptic transgenic R^+^A^+^ mothers and their offspring, and found evidence that supports the notion that mothers experience short-term deficits, while offspring showed longer lasting impairments on an object recognition task as far as 12-months of age. Moreover, we provide new evidence for alterations in cerebrovascular function in offspring at a time when they display cognitive deficits.

One link worth further exploring between altered cerebrovascular and behavioral function in offspring of preeclamptic mothers stems from the fact that levels of placental growth factor (PGF) were markedly low during a preeclamptic pregnancy. One group has used PGF knockout mice as a preclinical model for offspring of preeclamptic mothers and found working memory deficits along with increased vascular density and length at the capillary level, without any changes in cerebral blood volume (Kay et al., 2018). Furthermore, when challenged with a common carotid artery ligation, CBF was lower in the PGF knockout mice, as measured by laser Doppler flowmetry (Luna et al., 2016). Other factors implicated in preeclamptic pregnancies include a hypoxic fetal environment, increased exposure to cortisol, altered production of angiogenic factors, and increased production of pro-inflammatory cytokines, all of which could impact the development of the cerebrovascular system (Kay et al., 2019; Maher et al., 2019). Furthermore, epigenetic alterations have been described in the preeclamptic placenta, mostly in the context of disease development and associated to the environmental changes listed above. Hence, the possibility that abnormal DNA methylation during preeclamptic placental development is involved in long-term cognitive consequences merits further investigation (Apicella et al., 2019; Kamrani et al., 2019). While we acknowledge that there could be many factors at play that caused the offspring of preeclamptic mothers to show cognitive deficits, we explored the possibility that a consequence of these *in utero* alterations could be associated with altered cerebral hemodynamics, which have been found to underlay cognitive impairment in different contexts related to cardiovascular disease and dementia.

The regulation of CBF is altered by pregnancy, and even further by hypertension; both hypo- and hyperperfusion have been observed in preeclamptic mothers (Jones-Muhammad and Warrington, 2019), and this seems to be related to the severity of the condition, whereby more severe preeclampsia resulted in hyperperfusion measured in the middle cerebral artery by transcranial laser Doppler prior to delivery (Belfort et al., 1999; van Veen et al., 2015). Although we did not measure baseline perfusion, we found elevated whisker-evoked CBF responses and cerebral blood volume characterized by enhanced evoked relative oxygenated hemoglobin levels in 12 month-old offspring of R^+^A^+^ preeclamptic mothers. Interestingly, while we found an enhanced hyperemic response, hyperperfusion has been observed in preeclamptic patients and has been linked to decreased vascular resistance, which can further lead to disruptions of the blood brain barrier and negative neurological outcomes (Hammer and Cipolla, 2015). While the resistance in an individual capillary is quite high, when there is a dense capillary bed with several small vessels running parallel to one another, vascular resistance drops (Mandeville and Rosen, 2002). It is therefore possible that if the offspring in our study also experienced altered angiogenesis as seen in the PGF model (Kay et al., 2018), then global cerebral vascular resistance could be decreased, thus providing an explanation for the observed increased perfusion in response to whisker stimulation compared to control offspring. In a hypertensive environment, cerebral vessels are remodeled to protect against sudden spikes in blood pressure, however this could impair their general function at lower blood pressures (Jones-Muhammad and Warrington, 2019). Due to the offspring developing in a hypertensive environment and thus exposed to circulating factors that cross the placental barrier, despite the majority not being hypertensive themselves, this could also help to explain the elevated hemodynamic responses.

There exists a tight communication between neural cells and blood vessels termed neurovascular coupling, and here we observe an altered hemodynamic response whereby too much blood is being sent to the activated somatosensory cortex. We found that oxygenated hemoglobin levels were elevated in response to the same sensory stimulus in offspring of preeclamptic mothers. Capillaries are comprised mainly of endothelial cells, which play an important role in neurovascular coupling (Chen et al., 2014), and are the site of oxygen exchange, making it tempting to suggest that this is the primary site of dysfunction in the offspring, whereby either too much oxygen is being extracted into the parenchyma or that blood flow is too fast, and thus preventing oxygen extraction, leading to high levels in the blood. Either way, there is a clear lack of control over CBF that resulted in an elevated, unfocused/diffuse response in the parenchyma, and such hyperperfusion has been proposed as a compensatory mechanism in asymptomatic individuals at risk for late life dementia (Ostergaard et al., 2013).

In this pilot study we used the pups from an established model of preeclampsia and validated their use for future studies as a realistic model of offspring of preeclamptic mothers in terms of their cognitive outcome. Our findings are in line with cognition being altered in preeclamptic mothers in the short-term without any lasting deficits (Dayan et al., 2018; Elharram et al., 2018). They also draw attention to the necessity for more research focusing on children born under preeclamptic conditions to better understand the cerebrovascular mechanisms that may accompany cognitive deficits to try and establish treatments that could be used to actively counteract them from transpiring.

## Data Availability Statement

The raw data supporting the conclusions of this article will be made available by the authors, without undue reservation, to any qualified researcher.

## Ethics Statement

The animal study was reviewed and approved by the Animal Ethics Committee of the Montreal Neurological Institute.

## Author Contributions

LT assisted in the design of the study, conducted all behavioral experiments, measured blood pressure and took urine samples at all time points excluding baseline, analyzed data, and wrote and finalized the manuscript. CL implanted mice with cranial windows and performed optical imaging experiments and analysis. JR assisted in the study design and acquired baseline urine and blood pressure measurements. JL provided the mouse model used in this pilot study and provided equipment for urine analysis to assess proteinuria. FL provided their expertise and codes for analysis of optical. LP helping to initiated the study and contributed to the editing of the final manuscript. EH supervised the project, assisted in the design of the study, contributed to the writing, and editing of the final manuscript. All authors contributed to the article and approved the submitted version.

## Conflict of Interest

The authors declare that the research was conducted in the absence of any commercial or financial relationships that could be construed as a potential conflict of interest.
